# How Nanotechnology and Biomedical Engineering Are Supporting the Identification of Predictive Biomarkers in Neuro-Oncology

**DOI:** 10.3390/medicines5010023

**Published:** 2018-02-26

**Authors:** Mario Ganau, Marco Paris, Nikolaos Syrmos, Laura Ganau, Gianfranco K.I. Ligarotti, Ali Moghaddamjou, Lara Prisco, Rossano Ambu, Salvatore Chibbaro

**Affiliations:** 1 Department of Neurosurgery, Toronto Western Hospital, University of Toronto, Toronto, ON M5T 2S8, Canada; alim937@hotmail.com; 2 School of Medicine, University of Cagliari, 09124 Cagliari, Italy; lolly26it@yahoo.it (L.G.); amburo@unica.it (R.A.); 3 National Hospital for Neurology and Neurosurgery, University College London, London WC1N 3BG, UK; marco.paris@uclh.nhs.uk; 4 School of Medicine, Aristotle University of Thessaloniki, 54623 Thessaloniki, Greece; milanako76@yahoo.gr; 5 Fondazione IRCCS IstitutoNeurologico “Carlo Besta”, 20133 Milano, Italy; gianfrancokiligarotti@gmail.com; 6 John Radcliffe Hospital, Oxford University, Oxford OX3 9DU, UK; lara.prisco@ndcn.ac.ox.uk; 7 Division of Neurosurgery, University of Strasbourg, 67000 Strasbourg, France; schibbaro@hotmail.com

**Keywords:** biomarkers, precision medicine, innovation, oncology, neurosurgery

## Abstract

The field of neuro-oncology is rapidly progressing and internalizing many of the recent discoveries coming from research conducted in basic science laboratories worldwide. This systematic review aims to summarize the impact of nanotechnology and biomedical engineering in defining clinically meaningful predictive biomarkers with a potential application in the management of patients with brain tumors. Data were collected through a review of the existing English literature performed on Scopus, MEDLINE, MEDLINE in Process, EMBASE, and/or Cochrane Central Register of Controlled Trials: all available basic science and clinical papers relevant to address the above-stated research question were included and analyzed in this study. Based on the results of this systematic review we can conclude that: (1) the advances in nanotechnology and bioengineering are supporting tremendous efforts in optimizing the methods for genomic, epigenomic and proteomic profiling; (2) a successful translational approach is attempting to identify a growing number of biomarkers, some of which appear to be promising candidates in many areas of neuro-oncology; (3) the designing of Randomized Controlled Trials will be warranted to better define the prognostic value of those biomarkers and biosignatures.

## 1. Introduction

In quantitative neuroscience, identifying suitable biomarkers is pivotal to streamlining the clinical screening for early and ultra-early diagnosis of many diseases, including cancers. Biomarkers are quantitative biological signatures of any given physiological state or pathological condition, used in many areas of medicine to estimate the risk of developing specific diseases, the likelihood and rapidity of their progression, as well as the prediction of their outcome [1,2]. Biomarkers may be used individually or in combination: two or more biomarkers (i.e., a profile of data gathered from imaging, genomics and proteomics testing), in fact, are usually referred to as a biosignature. As a general rule, a composite measure, such as a biosignature, can significantly enhance the sensitivity and specificity of diagnostic protocols when compared to that of each measure alone [1,2].

As biomarkers became integrated into drug development, clinical trials and modern medicine, they gained the spotlight, becoming of preponderant importance in the continuous crosstalk between several stakeholders, including scientific and clinical community, multinational pharmacological companies, high-tech biomedical startups, investors, and obviously patients. Given the attention around their role, in recent years, a need for a shared understanding and a common language revolving around biomarkers has arisen. For instance, in early 2016, the Food and Drug Administration (FDA) and the National Institutes of Health (NIH) published the first version of the glossary included in the Biomarkers, EndpointS, and other Tools (BEST) resource, which was constructed to harmonize and clarify terms used in translational science and medical product development and to provide a common ground for communication among those agencies [3]. The BEST resource clearly classifies biomarkers according to their specific role into the following more homogeneous groups: susceptibility risk biomarkers, diagnostic biomarkers, monitoring biomarkers, prognostic biomarkers, predictive biomarkers, pharmacodynamic response biomarkers, and safety biomarkers. 

Each group of biomarkers intended for use in patient care undergoes a rigorous evaluation prior to introduction into the clinical practice; the analytical tests proposed to measure a candidate biomarker are no exception to this well defined process to assess their accuracy and reliability. Since the integration of various technologies is essential to innovation, and proved pivotal to not only biomarker identification and characterization but also validation, a great deal of attention has been recently put on quality assurance and, in particular, assay validation [4]. Similarly to what was done with the BEST resource, to add clarity to the language used by oncologists and basic scientists within the context of precision medicine, the “European Society of Medical Oncologists (ESMO) Translational Research and Personalised Medicine Working Group” has developed a standardized glossary of relevant terms [5]. This working group highlighted five main areas of interest: (1) mechanisms of decision, (2) characteristics of molecular alterations, (3) tumor characteristics, (4) clinical trials and statistics, (5) new research tools. Given the importance of the latter, in this systematic review we aim to summarize the impact of nanotechnology and biomedical engineering in defining clinically meaningful predictive biomarkers with a potential application in the management of patients with brain tumors. In particular, we will focus on the latest discoveries in quantitative neuroscience, specifically those that, are rapidly finding a place in modern clinical practice and therefore hold the promise to foster the field of personalized medicine in neuro-oncology.

## 2. Materials and Methods

This article aims at providing readers with an overview of all the most recent studies in which the role of new devices based on innovative discoveries coming from the field of nanotechnology and biomedical engineering were highlighted with regards to clinical and functional profiling in neuro-oncology. 

*Study Characteristics*: Given the research question outlined above, this article focuses on basic sciences and clinical studies that have exploited innovations in nanotechnology or biomedical engineering applied to genomics, epigenomics and proteomics to validate already existing biomarkers and biosignatures, or to identify new ones with the potential to predict clinical and surgical outcomes in patients with brain tumors (of any sort, primary and secondary brain tumors). While we have included any type of experimental paper (including studies on animal models), the following types of articles were excluded from this review: review articles, letters, editorials/commentaries, meeting abstracts, and books. 

*Information Sources*: A systematic search of MEDLINE, MEDLINE in Process, EMBASE, and/or Cochrane Central Register of Controlled Trials was conducted to identify relevant studies. 

*Search Strategy*: We developed a search strategy with a librarian who specializes in neuroscience research. The strategy was first developed in MEDLINE and then appropriately modified for the other databases. The following search terms were used at time of interrogating all databases (November 2017): “Brain Tumors” AND “Nanotechnology” or “Biomedical Engineering”, AND “biomarkers” or “biosignatures”, AND “clinical outcomes” or “surgical outcomes”. Only studies written in English were considered for inclusion, with no other limits applied in terms of type of study (basis science/clinical study). The results of this search were thoroughly reviewed: initially by four authors with extensive experience in basic laboratory studies, and finally validated by four authors with clinical expertise on management of brain tumors. A final check by all authors was carried out to ensure that only experimental studies providing: (a) a materials and methods section with a detailed description of new screening methods based on nanotechnology or biomedical engineering, and (b) a results section describing their correlation with clinical and surgical outcomes, had been retained for further analysis and report in this systematic review. 

*Data Extraction and Synthesis*: The following data were extracted from each included article: study design, publication date, samples used for identification of biomarkers/biosignatures, and clinical/surgical outcomes included in the study. Considering the heterogeneity of the studies included we decided that it would not have been appropriate to perform a meta-analysis. 

*Reporting*: The results of this review were formatted based on the Preferred Reporting Items for Systematic Reviews and Meta-Analyses (PRISMA) statement [6]. 

## 3. Results

**Figure 1:**
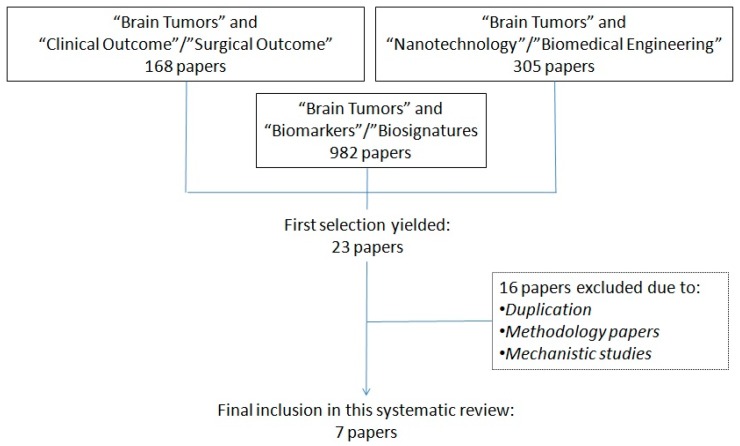
Selection process of suitable articles to be analyzed in this systematic review.

The initial search of the literature yielded to 1455 articles, which were then screened in two consecutive rounds by two groups of four experts (scientists for the first round and clinicians for the second round) involved in this study. This triage of the literature led to an initial selection of 23 papers, out of which 16 were excluded due to: duplication of the papers identified, or because the articles dealt with the description of a methodology or the description of physiological/pathological pathways, but eventually failed to provide a correlation between the identification of a biomarker/biosignature and the related clinical or surgical outcome. A diagram summarizing the design of this systematic review is provided (Figure 1). 

Depending on the technologies adopted for the identification of predictive biomarkers, the 7 articles eventually selected fall into two main categories: (a) MicroRNA, or (b) Multiplexing and Immunoassays. The 4 MicroRNA studies were all based on patients with gliomas (3 studies), or ependymomas (1 study); whereas the 2 Multiplexing and Immunoassays studies included cases of gliomas and meningiomas, respectively. Of note, only 1 study attempted to identify a biosignature on patients with gliomas by correlating a specific radiological pattern with the genetic profile of the tumor studied [7,8,9,10,11,12,13]. 

A summary for each of the 7 articles identified in this systematic review, including research setting, details of the cohort/sample studied, analytical methodology, biomarker(s) identified, most significant results and related externalities in term of prognosis, is provided in Table 1, Table 2, Table 3 and Table 4.

## 4. Discussion

Attempts to identify biomarkers and biosignatures to monitor the spread of neoplastic cells within the central nervous system (CNS) and predict the risk of recurrence of the disease following an initial medical and surgical treatment represent the latest frontier in neuro-oncology. The DIRECTOR trial in which authors prospectively assessed by methylation-specific polymerase chain reaction (PCR) the status of the *O*^6^-methylguanine DNA methyltransferase (MGMT) promoter, and correlated it with clinical response to adjuvant chemotherapy, is one of the best examples of how biomarkers could be exploited to guide the decision making process in a personalized way [14]. Given their potential to optimize the management of many primary and secondary brain malignancies, an increase in the pool of available biomarkers could potentially provide clinicians with a new pillar to support their surgical and medical choices. At present, only few biomarkers are approved by regulatory authorities for CNS Tumors. Traditional biomarkers include 1p/19q co-deletion, MGMT methylation, and mutations in IDH1/IDH2. According to the definitions extrapolated from the BEST resource they classify as diagnostic, prognostic and predictive biomarkers, and so are more recently tested biomarkers that could be isolated from the serum of cancer patients, such as: VEGFR-2, EGFRvIII, PGAM1, IL2, PDGFR, MMPs, BRAF, STAT3, PTEN, TERT, AKT, NF2, and BCL2 [15,16,17,18,19,20,21,22,23,24]. Excellent research articles and reviews cover not only the rationale, but also the clinical role of these biomarkers, and an additional discussion on them is beyond the scope of our research question. Nonetheless, it is worth mentioning that most of their clinical use is based on specific testing and methodologies, or relies on extrapolation of clinical data from other research on a common biomarker/biosignature [25]. Hence the relevance of our systematic review, which provides readers with the state of the art in terms of efforts to: (a) implement innovative methodologies, (b) effectively apply them in the area of neuro-oncology, and (c) define the prognostic value of the many biomarkers currently investigated.

*Identifying biomarkers in neuro-oncology*: The improved sensibility and sensitivity of modern biosensors has allowed molecular diagnostics to rapidly move beyond genomics to proteomics, and to identify a disease based on the related pathognomonic post-translational modifications [26]. The proteome and secretome, by definition, are dynamic, and change both in physiologic and in pathologic conditions; the ultimate goal of determining them is therefore to characterize the flow of information within the cells, through the intercellular protein circuitry that regulates the extracellular microenvironment. The tumor microenvironment represents a very complex and heterogeneous system, consisting of intricate interactions between the tumor cells and its neighboring non-cancerous stromal cells. This statement is particularly true for gliomas, the most common primary brain tumors, which happen also to be the more studied ones, as confirmed by the design of 5 out of 7 of the studies selected for this systematic review [8,9,10,11,13]. To delve deeper into this topic, it is worth mentioning that the tumor cells and their progenitor stem cells responsible for glioma progression show unique behaviors due to variation in several genetic and environmental factors. Similarly, the behavior of stromal cells involved in the tumorigenesis of other brain tumors (i.e., meningiomas, ependymomas, etc.) can have dramatic implications in the pathogenic conditions of those brain tumors. In both scenarios, identifying the markers of cancer stem cells represents the key to tackle the source for resistance to chemo- and radiotherapy, or again the relapse of those tumors despite an initially radical surgical resection. For these reasons, efforts in neuro-oncology, continue to focus on elucidating the complex molecular mechanisms underlying the pathophysiology of brain cancers, with the aim to define sensitive and specific biosignatures exploitable in clinical settings [27,28]. 

*The impact of nanotechnology and bioengineering*: A wide range of laboratory and consumer biotechnological applications, from genetic and proteomic analysis kits, to cell culture and manipulation platforms, allow in vitro analyses of established oncological biomarkers, and more generally enable scientists to predict the behavior of neoplastic cells under various exogenous stimuli [29]. Of note, point-of-care diagnostic testing, which makes it possible to test directly at the patient’s bedside, has the aim of enabling physicians to diagnose a patient’s conditions more rapidly than conventional lab-based testing. By using these devices to reduce the time to diagnosis, the physician is able to make better patient management decisions, leading to improved patient outcomes and reduce the overall cost of care. 

Of note, advances in microelectronics and biosensor tools were particularly instrumental in facilitating the development of these diagnostic devices. Various platforms were developed to allow for the simultaneous real-time evaluation of a broad range of disease markers by non-invasive techniques. Among them, two classes of microtechnological devices developed since the early 1990s, microarray DNA chip and microfluidics systems for lab-on-a-chip diagnostics, have found their full application following further miniaturization at the nanoscale [30]. Several techniques from the field of nanotechnology are nowadays available for the miniaturization and biofunctionalization of diagnostic surfaces, with promising results in the screening armamentarium for molecular analysis. Indeed, many of them appear particularly suitable for a high-sensitivity determination of panels of biomarkers.

*Multiplexing and Immunoassays*: Highlighting the issue of inter- and intra-tumor heterogeneity, many single-cell analysis techniques, such as cell-based, nucleic acid-based, protein-based, metabolite-based and lipid-based, emerged over the last decade as an important approach to detect variations in morphology, genetic or proteomic expression within the tumor niche [30]. The demand for parallel, multiplex analysis of protein biomarkers from very small biospecimens obtained at time of surgery, or through blood/CSF samples during follow-up, represented for years an increasing trend, mostly aimed at creating spatially encoded microarrays able to capture multiple proteins of interests in a cell lysate all at once [29]. For instance, in one of the articles identified by this systematic review, the gene expression levels of NANOG, a key regulator of pluripotency, and therefore a marker of stem cell-like behavior, was quantitatively tested along with other proteins (SOX2, OCT4, KLF4, ABCG2, CMYC, MSI1, CD44, NOTCH1, NES, SALL4B, TP53, and EPAS1) using Real Time-quantitative PCR (RT-qPCR) in 33 surgical specimens of low- (WHO grade I) as well as in high-grade (WHO grade II/III) meningiomas [12]. Additionally, immunofluorescence co-localization analysis following confocal fluorescence microscopy for NANOG, OCT4, SOX2, Nestin, KI-67, and CD44 was also performed. These techniques made it possible to support the research theory that an overexpression of NANOG and other markers of pluripotency and stemness in meningiomas, such as SOX2 and OCT4, could be exploited to target potentially pluripotent "stem cell-like" cells [12]. Those NANOG-positive cells seem to be only 1% in low-grade, and 2% in grade II/III meningiomas; nonetheless, in this study and in other literature, they have demonstrated their remarkable impact on tumorigenesis and progression in human meningiomas and high-grade gliomas, being correlated with the overall clinical and surgical prognosis [12,31,32,33].

*miRNA in bioengineering*: microRNAs (miRNAs) are small RNAs 18 to 24 nucleotides in length that serve the pivotal function of regulating gene expression. Instead of being translated into proteins, the mature single-stranded miRNA binds to messenger RNAs (mRNAs) to interfere with the translational process. It is estimated that, whereas only 1% of the genomic transcripts in mammalian cells encode miRNA, nearly 1/3 of the encoded genes are regulated by miRNA. Experimental studies on miRNA provide a new bioengineering approach for understanding the mechanism of fine-tuning gene regulation [34]. The growing interest for this methodological approach is testified by the fact that 5 articles identified in our review revolve around the identification and analysis of miRNA and mRNA. 

Steponaitis et al. investigated the mRNA expression of Chitinase 3-like 1 (CHI3L1), a protein playing multiple roles in cell proliferation, differentiation, apoptosis, angiogenesis, inflammation and extracellular tissue remodeling; whereas Vaitkienė et al. studied the mRNA levels of semaphorin 3C (Sema3C), a protein involved in tumorigenesis and infiltration of extracellular matrix [10,11]. Both studies managed to confirm the role of those biomarkers in predicting the outcome of patients and their response to adjuvant treatment, therefore those groups concluded that their findings could be particularly relevant in defining novel treatment strategies against gliomas. 

As opposed to studies on mRNA, which are usually very specific, those on miRNA provide information about many more different genes. For instance, the three articles reported in Table 1 exploiting this methodology investigated a total of 22 different miRNA selected from a much larger pool of those initially considered by the authors at time of screening. Furthermore, the information obtained from studies on miRNA seem to be particularly useful at time of complementing them with those achievable with multiplexing or immunoassay proteomic studies [7,8,9]. This was the case also for the only study, among the selected ones, which resulted in successfully determining a biosignature of WHO Class II and III gliomas. Kickingereder et al., in fact, demonstrated that the gene expression and mutations of hypoxia-inducible-factor 1-alpha (HIF1A), a driving force in hypoxia-initiated angiogenesis, can be non-invasively predicted with a specific MRI sequence, known as relative cerebral blood volume (rCBV) imaging [13]. Considering that only 25 articles have been published so far on the radiogenomics of brain tumors, and that most of them are review articles, this study certainly classifies as a landmark paper: the ability to predict with imaging, and therefore in a fast and non-invasive way, a distinct angiogenesis transcriptome signature pinpoints the remarkable advancement that proteomic profiling is achieving, thanks to the conjunction between innovative technologies and bioengineering. 

*Future applications*: Despite the fact that we had not set a specific timeframe for our initial research, and therefore considered all available articles regardless of date of publication (the oldest ones dated back to 1983), those eventually fulfilling our inclusion criteria turned out to be very recent, having all been published between 2015 and 2017. Not surprisingly, similar findings have also been pinpointed in other systematic reviews on neuro-traumatology carried out by our research team [35,36]. Indeed, the ongoing research efforts outlined in our review suggest two directions for future development of detection tools, and the translation of their use from bench to bedside: (1) a more efficient clinical monitoring of progression of brain tumors through well-defined biosignatures, and (2) a strict surveillance of emerging drug-resistant cells following chemo- and radiotherapy through pharmacodynamic response and safety biomarkers. As such, the more meaningful goal of molecular biomarker discovery will be to help clinicians and surgeons in formulating faster and more accurate diagnostic and therapeutic management plans [37,38]. One last externality of these efforts in proteomic profiling is the impact on conventional neuroimaging, which is meant to benefit from the molecular insights highlighted above [39,40,41,42]. In fact, innovative nanocarriers for contrast agents are opening the path toward enhanced recognition/imaging definition of brain and spinal pathologies at a cellular level, while serving, as demonstrated in other fields, also for theranostics purposes [43,44,45,46,47,48,49].

*Limitation of this systematic review*: This article on neuro-oncology was aimed at describing the state of the art in the application of nanotechnology and biomedical engineering in providing clinically meaningful biomarkers with a potential to improve the management of patients with primary and secondary brain tumors. Therefore, the design of this systematic review did not include additional searches of the grey literature (including abstract or conference proceedings) to avoid the bias of including studies not validated by an external thorough peer review process. Also, we decided not to search other databases such as: (1) clinicaltrial.gov, (2) United States Patent and Trademark Office Database, and (3) European Patent Office Databases. This choice implied our inability to identify patents not referenced yet in the literature, or collect other data that could better show where the translation of basic research is heading. For instance, as a result of this decision we failed to identify which countries are investing the most or are lined up to obtain the highest return on their investments in nanotechnology and biomedical engineering. Whereas we estimate a crossover for many articles that would have been identified through those additional searches, and therefore we believe that we have not lost any relevant scientific information, this limitation was identified and accepted early on in the initial stages of our study, and eventually deemed not relevant for the research question clearly stated at the beginning of this article.

## 5. Conclusions

Indeed, the study of proteomics and molecular biomarkers in neuro-oncology has already made it possible to identify direct or indirect predictive factors, and to determine which affected pathway has more chance of being a selective therapeutic target. Those driving forces are allowing life-science researchers worldwide to unravel the mechanisms involved in development of brain tumors, and decipher the molecular characteristics of these malignancies. Based on the results of this systematic review, which screened over 1455 articles, we can conclude that: (1) the advances in nanotechnology and bioengineering are supporting tremendous efforts in optimizing the methods for proteomic profiling, (2) a successful translational approach is making it possible to identify a growing number of biomarkers that appear to be promising candidates in many areas of neuro-oncology, (3) the natural step of designing Randomized Controlled Trials will consequently be warranted to better define the prognostic value of those biosignatures. Should those trends continue, it can be easily forecasted that approved protocols that implement all those discoveries will herald a new era of precision and personalized neuro-oncology.

## Figures and Tables

**Table 1:** Nanotech and Biomedical Engineering advances in identification of MicroRNA biomarkers in neuro-oncology.

Methodologies and References	Tumor Histology	Findings
*MicroRNA*		
Margolin-Miller, Y.; et al., 2017 [7]	Pediatric Ependymoma (WHO Class II–III)	Following miR-array expression analysis, 9 miRNAs that correlated with relapse of disease were further validated by quantitative real-time PCR in a cohort of 67 patients. Eventually, miR-124-3p emerged as an independent prognostic factor of relapse. Negative levels of the miR-124-3p target (protein TP53INP1) also correlated with a poor outcome.
Schliesser, M.G.; et al., 2016 [8]	Anaplastic Glioma (WHO Class III)	Out of 12 putative miRNA promoter regions identified from unbiased DNA methylation screens, miR-155 promoter methylation and miR-155 expression were demonstrated to have the strongest negative correlation with patient survival. MiR-155 also conferred resistance towards alkylating temozolomide and radiotherapy as consequence of nuclear factor (NF)κB activation.
Tang, H.; et al., 2015 [9]	Glioma (WHO Class III–IV) Meningioma, Pituitary Adenoma and Acoustic Schwannoma (WHO Class I–II)	Plasma levels of miR-185 results significantly altered in glioma patients compared to normal controls; of note, low plasma levels seems to correlate with poor survival. Of note, miR-185 levels do not appear observably changed in patients with other brain tumors, such as meningioma, acoustic schwannoma, or pituitary adenoma. Furthermore, in Grade IV gliomas treated with surgical excision and chemo-radiotherapy, the plasma levels of miR-185 almost returned to normal levels.

**Table 2:** Nanotech and Biomedical Engineering advances in identification of mRNA biomarkers in neuro-oncology.

Methodologies and References	Tumor Histology	Findings
*mRNA*		
Steponaitis, G.; et al., 2016 [10]	Glioma (WHO Class I–IV)	CHI3L1 expression was assessed with quantitative real-time PCR in a cohort of 98 patients: mRNA expression of CHI3L1 was evidently higher in glioblastoma than in lower-grade glioma tissues. However, patients with high CHI3L1 expression had a shorter overall survival regardless of their histology (high-grade as well as lower-grade gliomas).
Vaitkienė, P.; et al., 2015 [11]	Glioma (WHO Class I–IV)	Protein and mRNA levels of semaphorin 3C (Sema3C), a protein involved in tumorigenesis and metastasis were studied in a cohort of 84 patients. Protein levels markedly increased in grade IV gliomas compared to grade I–III astrocytomas and were significantly associated with the shorter overall survival of patients. Sema3C mRNA levels showed no association with either grade of glioma or patient survival.

**Table 3:** Nanotech and Biomedical Engineering advances in Multiplexing and Immunoassays techniques for biomarkers in neuro-oncology.

Methodologies and References	Tumor Histology	Findings
*Multiplexing and Immunoassays*		
Freitag, D; et al., 2017 [12]	Meningioma (WHO Class I–III)	The overexpression of NANOG, a key regulator of pluripotency and malignant behavior, was studied by single-cell immunoassay in a cohort of 33 patients. While low-grade meningiomas expressed 1% NANOG+ cells, the rate rose to 2% in grade II/III meningiomas. Of note, NANOG+ cells also expressed other markers of pluripotency (i.e., SOX2 and OCT4), thus being demonstrated to act as “stem cell-like” cells with an impact on tumorigenesis and progression.

**Table 4:** Nanotech and Biomedical Engineering advances in identification of biosignatures in neuro-oncology.

Methodologies and References	Tumor Histology	Findings
*Biosignature—MicroRNA plus MRI features*		
Kickingereder, P.; et al., 2015 [13]	Glioma (WHO Class II–III)	A genotype/imaging phenotype correlation analysis with relative cerebral blood volume (rCBV) MRI, a robust and non-invasive estimate of tumor angiogenesis, showed in a cohort of 73 patients that a one-unit increase in rCBV corresponds to a two-thirds decrease in the odds of an IDH mutation and correctly predicts IDH mutation status in 88% of patients. Given the role of IDH gene in hypoxia-inducible-factor 1-alpha (HIF1A), a driving force in hypoxia-initiated angiogenesis, this study demonstrated that IDH mutation status, and the associated distinct angiogenesis transcriptome signature, can be non-invasively predicted with rCBV imaging.
